# Forms and Migration Mechanisms of Phosphorus in the Ice, Water, and Sediments of Cold and Arid Lakes

**DOI:** 10.3390/toxics12070523

**Published:** 2024-07-20

**Authors:** Weiying Feng, Yingru Tao, Tengke Wang, Fang Yang, Meng Zhao, Yuxin Li, Qingfeng Miao, Tingting Li, Haiqing Liao

**Affiliations:** 1 School of Materials Science and Engineering, Beihang University, Beijing 100191, China; fengweiying@buaa.edu.cn (W.F.); taoyingru2000@163.com (Y.T.); zy2130206@buaa.edu.cn (T.W.); 2 State Key Laboratory of Environmental Criteria and Risk Assessment, Chinese Research Academy of Environmental Sciences, Beijing 100012, China; litingting193@163.com (T.L.); liaohq@craes.org.cn (H.L.); 3 Institute of Plant Nutrition, Resources and Environment, Beijing Academy of Agriculture and Forestry Sciences, Beijing 100097, China; 4 College of Basic Medical Science, Inner Mongolia Medical University, Inner Mongolia Key Lab of Molecular Biology, Hohhot 010059, China; 20200043@immu.edu.cn; 5 College of Water Conservancy and Civil Engineering, Inner Mongolia Agricultural University, Hohhot 010018, China; imaumqf@imau.edu.cn; 6 State Key Joint Laboratory of Environment Simulation and Pollution Control, School of Environment, Tsinghua University, Beijing 100084, China

**Keywords:** ice, lake, aquatic ecosystem, ^31^P-NMR, freeze–thaw cycle

## Abstract

Phosphorus (P) is a crucial nutrient in lake ecosystems and organic phosphorus (Po) is a significant component. However, the distribution characteristics and migration behaviour of Po in ice–water–sediment systems under freezing and thawing conditions in cold and arid regions remain unclear. This study aims to investigate the forms of Po and its contribution to endogenous P pollution. We selected three lakes (Dai, Hu, and Wu Lake) and employed phosphorus nuclear magnetic resonance (^31^P-NMR) techniques to analyse the following: (1) The total phosphorus (TP) content, which was the highest in the water from Dai Lake (0.16 mg/L), with substantial seasonal variation observed in Wu Lake, where P content was four times higher in summer than in winter because of farmland drainage. (2) Eutrophication analysis, which indicated that Dai Lake had significantly higher eutrophication levels than Wu Lake, with P being the controlling factor in Dai Lake and both N and P in Wu Lake. The proportion of Po in the TP content was 90%, 70%, and 55% for Wu, Dai, and Hu Lake, respectively, indicating that Po was the main component of eutrophic lakes. (3) ^31^P-NMR, which revealed that orthophosphate (Ortho-P) and monoester phosphate (Mon-P) were the main P components in the winter, with a higher P content in Dai Lake. Ortho-P has a higher content in ice, indicating that inorganic phosphorus (Pi) migration is the main factor in ice–water media. Mon-P showed multiple peaks in Dai Lake, indicating a complex composition of adenosine monophosphate and glucose-1-phosphate. (4) The ice–water phase change simulation experiments, which showed that phosphate was the least repelled in ice, while pyrophosphate (Pyro-P) and macromolecular P were more repelled. Adding sediment enhanced the migration of P but did not change the repulsion of macromolecular P, suggesting the molecular structure as the main influencing factor. These results provide important scientific evidence for the quantitative analysis of Po pollution in lake water environments, aiding in P load reduction and risk prevention and control.

## 1. Introduction

Lakes in cold and arid regions worldwide are increasingly affected by climate change and human activities, resulting in shrinking lake areas, water resource shortages, deteriorating water environments, and degraded water ecosystems [1,2]. These changes severely impact the health of natural ecological environments and the safety of domestic water supplies. Addressing the complex interactions of multiple influencing factors, diverse pollution sources, and the recurring nature of environmental governance in these regions is crucial. Understanding the migration and transformation mechanisms of phosphorus in different media (ice, water, and sediment) during the icebound period has emerged as a critical issue that requires urgent attention [3]. Most studies on lake eutrophication focus on the non-icebound period, whereas this study takes a unique approach by focusing on the distribution and migration of different phosphorus components in lakes during the winter icebound period [4]. There are relatively few studies on this period; however, it is crucial for a comprehensive understanding of the annual phosphorus cycle and eutrophication process in lakes [5,6].

Phosphorus (P) is a fundamental element in life processes, crucial for DNA structure energy transfer, and is a key nutrient in lake ecosystems [7,8,9]. In recent decades, P pollution in water bodies has remained high [10]. P includes inorganic phosphorus (Pi) and organic phosphorus (Po), with orthophosphate (Ortho-P) being the Pi that can be directly utilised by organisms [11]. The distribution, form, utilisation mechanism, migration, and transformation mechanisms of Pi in different media have been widely studied [12,13,14,15,16]. Po, as a bioavailable P source for organisms such as cyanobacteria, is a key factor in lake eutrophication [17,18,19,20].

Lake eutrophication is mainly affected by two factors. First, the inflow of eutrophic water from rivers and other sources into lakes gradually increases eutrophication. For example, the impact of farmland drainage on the P content and nitrogen/phosphorus ratio (N/P) in lakes is significant, as centralised planting leads to the inflow of nitrogen and P into lakes from the soil [21]. The second factor is the enrichment of nutrients within the internal ecosystem of lakes, leading to increased eutrophication. Research indicates that arid conditions can cause severe stratification of lake water quality and rising water temperatures, which, combined with algae proliferation, affect dissolved oxygen levels and water quality [22]. Understanding the dynamics of endogenous and exogenous Po in lakes during eutrophication is crucial for lake ecosystem protection [2,23,24].

Phosphorus affects the ecosystem structure through primary productivity, and excessive P can lead to lake eutrophication and algae blooms. Some algae release algal toxins that can harm the health of aquatic organisms and cause water pollution, posing risks from unclean water [25]. Po, as a potentially available component of P, includes monoester phosphorus (Mon-P), diester phosphorus (Di-P), and phosphonates, with dissolved organic phosphorus (DOP) and particulate organic phosphorus (POP) primarily composed of Mon-P accounting for over 40% of the total phosphorus (TP) [26]. Unlike other P components, Po is involved in photochemical reactions, releasing hydroxyl radicals (·OH) that increase soluble phosphate in water, exacerbating lake eutrophication [27,28]. Po can also undergo hydrolysis under alkaline conditions, making it a potential source of bioavailable P [29]. As a major component of aquatic plants [30], algal fragments in lakes are a major source of sediment Po pollution, and the accumulation of Po in sediments, combined with humus and humic acid, is an important source of active organic phosphorus in lakes [20]. Additionally, substances such as Po, polyphosphate, and pyrophosphate in algae can be released into the water through chemical and biological degradation, further aggravating lake eutrophication [3]. This in-depth analysis of specific P forms, such as Mon-P, provides a new perspective for understanding the role of Po in lake eutrophication.

This study aims to analyse the distribution of different P components in lakes during ice-covered periods through simulated experiments, focusing on the impact of P components on eutrophication. Investigating the composition and migration processes of Po in lakes during winter can help us understand the causes of eutrophication and provide insights into addressing this issue from the perspective of Po sources and migration. A combination of simulation experiments and field observations improves the reliability and practicality of research results. This method can more accurately reflect the actual situation and provide strong support for theoretical research and practical applications. In addition, a new phosphorus transfer mechanism between ice and water during the icebound period was proposed. By exploring the phosphorus transfer mechanism, especially Po, at the interface between ice and water during the icebound period, this study provides new insights for controlling the phosphorus load and reducing the risk of algal outbreaks. This can aid in developing more effective strategies for preventing and controlling algae blooms.

## 2. Materials and Methods

### 2.1. Study Area

**Figure 1:**
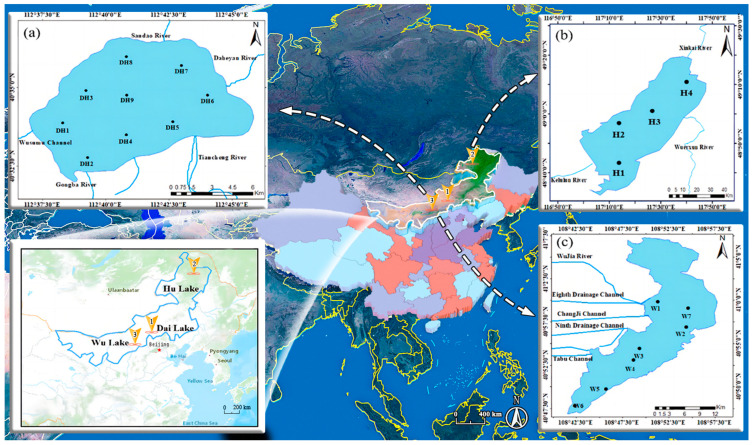
Schematic diagram of the geographical location and sampling points of lakes. (**a**) Dai Lake (**b**) Hu Lake (**c**) Wu Lake. (Black dots: sample sites; Yellow arrows: name of lakes).

Samples were collected from Dai Lake (N 40°29′27″–40°37′6″, E 112°33′31″–112°46′40″) in Wulanchabu City, Inner Mongolia, China [31]; Wu Lake (N 40°47′46″–40°59′47″, E 108°42′19″–108°54′33″) in Bayannur City [32]; and Hu Lake (N 48°30′40″–49°20′40″, E 117°00′10″–117°41′40″) in Hulunbuir City [33]. The lake area of Dai Lake, Wu Lake, and Hu Lake is 70, 293, 2315 km^2^, respectively; the mean water depth of Dai Lake, Wu Lake, and Hu Lake is 9 m, 1 m, 5.7 m, respectively [31,32,33]. Dai Lake is an inland lake in the farming and pastoral interlaced region, and the over-exploitation of water resources has caused the water surface to shrink rapidly, causing the extinction of large fish [2]. Wu Lake is a lake of agricultural irrigation and water withdrawal type, which is heavily eutrophic and polluted by the drained water into the lake; the water level of the Yellow River is receding [23]. Hu Lake is a grazing area grassland throughput lake with a small water inflow, deteriorating water quality, and degraded water ecology [33]. In response to these common and individual issues, this study selected these three different types of lakes as the research objects. Winter samples (ice, water, and sediments) and summer samples (water and sediments) from three lakes were collected on 12 January 2022 and 3 August 2022, respectively. Table S1 lists the atmospheric and water temperatures of the three lakes in two seasons. The sampling points are shown in Figure 1. Nine, four, and seven sampling points were collected from Dai, Hu, and Wu Lakes, respectively, totalling twenty sampling points (Figure 1). Ice, water, and sediment samples were collected from all points, with three repetitions for each medium at each sampling point, resulting in a total of 180 samples.

### 2.2. Sample Collection and Pretreatment

Ice samples (30 kg) were collected and placed in sterile sampling buckets for melting [2]. The melted samples were filtered through a 0.45 μm filter membrane, preserving the filter membrane for P component detection, and in particular, the detailed components of Po with ^31^P NMR [11], with the remaining filtered sample used for physicochemical property testing. Water samples were collected using a water sampler at a depth of 0.5 m [31], transferred to sterile sampling bottles, and sealed for laboratory analysis.

Sediment samples from three lakes were collected using a grab-bucket sediment sampler (Beijing, China, Zhongke Zhiyi Environmental Technology Company). A 20 g sediment sample was collected, frozen in a sterile plastic bag, and transported to the laboratory for physical and chemical property measurements. Sediment samples were freeze-dried using a freeze-drying machine (Scientz-12N, Ningbo Scientz Biotechnology Co., Ltd., Ningbo, China), ground and sieved through an 800-mesh sieve to obtain sediment powder (0.5 g). The sediment powder was mixed with ultrapure water (1:60 ratio) and extracted at 20 °C for 16 h. The supernatant was filtered through a 0.45 μm mixed cellulose ester membrane (Whatman ME25, Cytiva, Marlborough, USA), and the extracted solution was subjected to physical and chemical property testing.

### 2.3. Determination Method of Water Quality Indicators

TP was determined by the harmonised and validated SMT method developed by the European Committee for Standardization [26]. Pi was determined using the phosphomolybdenum method [34]. The concentration of Po was calculated by subtracting Pi from TP. Total organic carbon (TOC) was measured using a TOC analyser (Multi C/N 2100, Jena, Germany), and the detection limit was 4 μg/L. Total nitrogen (TN) was determined using the alkaline potassium persulfate ultraviolet spectrophotometric method [26,30]. When measuring the water depth, a homemade buoy-type detector connected to a 15 m measuring rope with a precision of 1 cm was employed. For calibration purposes, two predefined depths were utilised; utmost stability was maintained throughout the measurement process to ensure accuracy.

### 2.4. Determination of P Components in Ice and Water Using ^31^P-NMR

The particulate powder from melted ice and water samples was dissolved in a mixed solution of NaOH and EDTA, followed by ultrasonic oscillation at 25 °C for 30 min [26]. The extraction efficiencies are listed in Table S2. The extraction solution was centrifuged at 10,000 rpm for 30 min, and the supernatant was filtered and retained. A portion of the supernatant was added to 0.1 mL D_2_O to lock the signal and transferred to a 5 mm nuclear magnetic tube (WILMAD, Vineland, New Jersey, USA) for detection [26]. The cycle delay was set to D_1_ = 5 s, the number of scans was 24,000, the nuclear magnetic resonance frequency of ^31^P was 161.98 Hz, and the test temperature was 20 °C. Using a 1 mg/L standard solution of Ortho-P, the sample was analysed qualitatively and quantitatively with MestReNova software version 14.2.0 [26].

Table S2 shows the concentration of particulate P in the sample and the extraction efficiency using NaOH and EDTA. The use of a two-step concentration extraction of P indicates that more than 50% of the particulate P can be extracted. The P content of each component of the extraction solution in the ^31^P NMR spectrum represented the composition of P in the sample. The average extraction rates of P from the water and ice samples were 61.27% and 52.94%, respectively.

### 2.5. Multi-Media Migration Simulation Experiment for Different Types of P Components during the Freezing Process of Lakes

To investigate the migration of P at the ice–water interface during the freezing process of lakes, this study used ectopic cultivation to simulate the migration of P in ice and water during the freezing period. The simulation was divided into two groups: Group A contained only ice and water media, whereas Group B retained the original microorganisms in the ice, water, and sediment systems. This setup aimed to explore the migration process of different P components at the ice–water interface under consistent environmental conditions and to assess the role of microorganisms in this migration.

To maintain consistent experimental conditions, sediment was collected from point W7 in Wu Lake during winter, which was located in the middle of the lake, an area less affected by external material input. The water (400 mL) used in the simulation was ultrapure water (Dura, MilliporeSigma, Burlington, MA, USA) with a resistivity of 18.2 MΩ·cm, packed in 750 mL polyethylene plastic bottle. After freezing for 8 h, the ice was covered with ultrapure water to ensure that only the added P components were present in their initial state. The added P components were four groups of P standard substances: Ortho-P standard solution (CAS No. 7664-38-2), pyrophosphate (CAS No. 7758-16-9), inositol hexaphosphate (CAS 83-86-3), and triphenylmethyl phosphine (CAS No. 31158-32-4); these P standard substances were purchased from Sigma-Aldrich, St. Louis, MO, USA. Ortho-P and pyrophosphate are typical inorganic phosphorus compounds, whereas inositol hexaphosphate and triphenylmethyl phosphine are typical organic phosphorus compounds. They are widely present in lakes; therefore, these four representative phosphorus components were selected for the simulation experiment. The control group contained ultrapure water (400 mL) without any added P component. After 28 days of cultivation at temperatures ranging from −4 °C to 0 °C, the TP content in the simulated ice and water samples was measured using the harmonised and validated SMT method developed by the European Committee for Standardization [26]. The detailed settings for the experimental and control groups were as follows:

Group A: ice and water media only; A1: blank sample, ultrapure water frozen ice + ultrapure water; A2: A1 + 0.1 mg/L Ortho-P standard solution; A3: A1 + 0.1 mg/L sodium pyrophosphate (Pyro-P); A4: A1 + 0.1 mg/L inositol hexaphosphate; and A5: A1 + 0.1 mg/L triphenylmethyl phosphine. Group B: ice, water, and sediment (100 g) with microorganisms, blank sample; B1: A1 + original sediments at the bottom; B2: B1 + 0.1 mg/L Ortho-P standard solution; B3: B1 + 0.1 mg/L sodium pyrophosphate (Pyro-P); B4: B1 + 0.1 mg/L inositol hexaphosphate; and B5: B1 + 0.1 mg/L triphenylmethyl phosphine.

### 2.6. Data Processing and Analysis

The concentration of TP in the three lakes was the concentration sum of Pi and Po. The histogram was drawn by software OriginLab 2022b. Box plot of TOC/TP and TN/TP in lakes and line chart of simulation experiment were drawn by OriginLab 2022b. Spearman correlation coefficients were obtained at *p* < 0.05. ^31^P NMR spectrum of P in lakes’ ice and water was measured using MestReNova software version 14.2.0.

## 3. Results and Analysis

### 3.1. Analysis of Phosphorus Content in Lake Ice and Water

**Figure 2:**
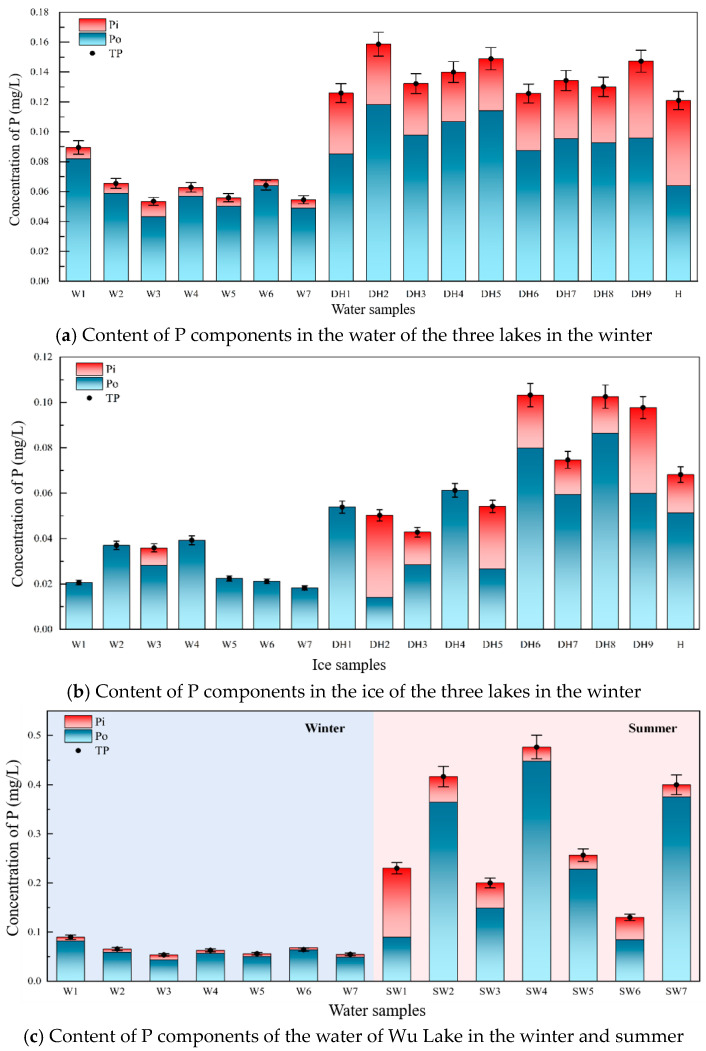
The content of P components in the lakes (W1–W7 water samples from Wu Lake, DH1–DH9 water samples from Dai Lake, H water sample from Hu Lake).

The TP, Pi, and Po contents in the water bodies of the Wu, Dai, and Hu lakes during winter are shown in Figure 2a. The results indicate that the TP content in Wu Lake ranges from 0.05 to 0.09 mg/L, in Dai Lake from 0.12 to 0.16 mg/L, and in Hu Lake is approximately 0.11 mg/L. The TP content in the water follows the order Dai Lake > Hu Lake > Wu Lake. The proportion of Po in the TP content is 90%, 70%, and 55% for the Wu, Dai, and Hu lakes, respectively, showing that Po constitutes a higher proportion of TP in Wu Lake than in the Dai and Hu lakes. This variation in P content and composition among the lakes can be attributed to their respective background environmental conditions. The high ratio of Pi to Po in Hu Lake suggests that nutrients are readily available for utilisation in the water during winter, possibly due to the inflow from surrounding livestock and other animals.

The concentrations of TP, Pi, and Po in the ice during winter for the Hu, Dai, and Wu lakes are shown in Figure 2b. The TP content in the ice mirrors that in the water, following the trend Dai Lake > Hu Lake > Wu Lake. The Pi content in the ice of Wu Lake is low, and the high detection limit of the instrument hindered the detection of Pi, except at point W3. Therefore, most ice samples primarily contained Po. Significant differences in P components were observed in the ice of Dai Lake at various sampling points. The P content was lower in the southwest and higher in the northeast, which aligns with the shrinking area of Dai Lake [35], indicating that environmental degradation is exacerbating the P content and contributing to lake eutrophication.

To better compare seasonal changes in P content, we examined Wu Lake’s water bodies in the winter and summer (Figure 2c). In the summer, the TP content in Wu Lake is approximately four times higher than in the winter. This increase is due to lush summer vegetation, optimal temperatures for algal metabolism, and inflows from farmland drainage. In the summer, Po constitutes approximately 80% of the TP in Wu Lake. The TP content also varies between different points in Wu Lake during the summer, with points W2, W4, and W7 showing approximately twice the TP content of other points. These three points are located where farmland drainage enters the lake, indicating that summer farmland drainage significantly impacts the P content in the lake. Farmland drainage introduces residual Po pesticides and other Po components into the lakes [36]. Therefore, managing lake P content must address the control of farmland drainage.

### 3.2. The Contribution of Lake Phosphorus Component Content to Eutrophication

Analysing the ratio of carbon, nitrogen, and P helps determine which element controls the nutritional status of a lake and provides strategies for controlling eutrophication. TOC reflects the total amount of organic matter, while total nitrogen (TN) indicates the total content of nitrogen-containing substances. The TOC and TN values of Dai and Wu lakes are listed in Table S3. From the TOC content, it can be seen that the organic matter content in the water is significantly higher than that in ice. In Wu Lake, the TOC in the water is approximately 5–10 times higher than in ice, and in Dai Lake, it is 5–7 times higher. Comparing both lakes, Dai Lake has higher levels of TOC, TN, and TP, indicating a more severe eutrophication problem than Wu Lake.

**Figure 3:**
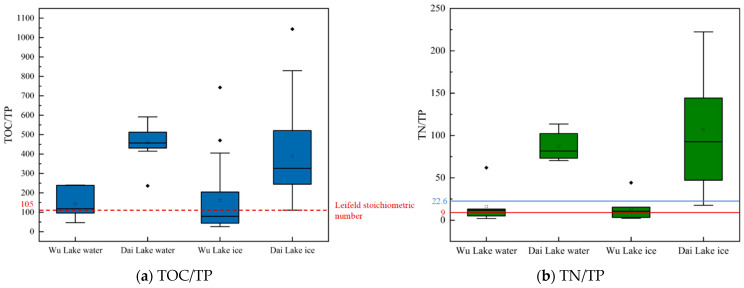
TOC/TP and TN/TP in lakes’ ice and water. (represents significance. Less than the solid red line represents the nitrogen limitation, and more than the solid blue line represents the phosphorus limitation, and the dotted red line is Leifeld stoichiometrc number).

The TOC/TP ratio reflects the eutrophication state, while the Leifeld stoichiometric number indicates potential nutrient proportions in the water [37]. Figure 3a shows the TOC/TP values for ice and water in the Wu and Dai Lakes during the winter. In Wu Lake, the TOC/TP values range from 52 to 247 in water and 38 to 389 in ice. In Dai Lake, these values range from 402 to 612 in water and 103 to 789 in ice. This indicates higher eutrophication in water than in ice, with Dai Lake exhibiting higher eutrophication levels than Wu Lake. The existing literature on Leifeld stoichiometric comparison confirms eutrophication in both the winter ice and water of the two lakes.

The N/P ratio indicates nutrient control over eutrophication and blue–green algal blooms. According to existing research, N/P < 9 suggests nitrogen limitation, 9 < N/P < 22.6 indicates dual control of nitrogen and P, and N/P > 22.6 suggests phosphorus control [30]. The N/P ratios for the Dai and Wu lakes in winter ice and water are shown in Figure 3b. In Wu Lake, the N/P ratio ranges from 9 to 22.6, indicating dual nitrogen and P control in winter. This indicates that nitrogen and P inputs during winter can lead to eutrophication. In contrast, the N/P ratio of Dai Lake in winter water and ice exceeds 22.6, indicating that the eutrophication of Dai Lake was mainly controlled by P. When P sources are added, blue–green algal blooms can occur. Seasonal analysis suggests that when lake ice melts and plants secrete P-containing substances, the eutrophication in Dai Lake intensifies [38]. The differences in nutrient control between the Wu and Dai lakes are related to their background values and environmental conditions, offering insights for addressing blue–green algal blooms in various lakes.

### 3.3. Analysis of the Composition and Structure of Po in Lakes

**Figure 4:**
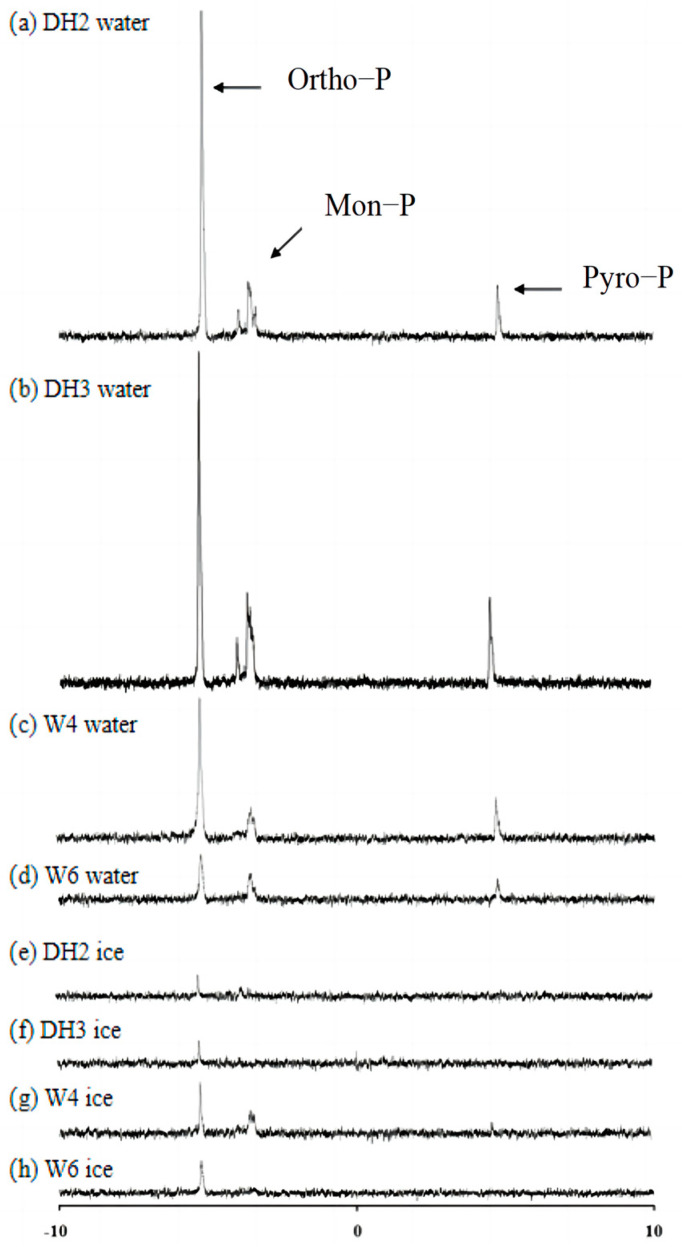
^31^P NMR spectra of phosphorus in lakes ice and water.

The components of P were identified using ^31^P NMR spectroscopy, as shown in Figure 4. The spectra indicate that the particulate P components in lake ice and water during winter mainly consist of Ortho-P and Mon-P, with Pyro-P components also present in the lake water. Ortho-P, an inorganic Pi component, dominates the exchange between lake ice and water in winter, reflecting Pi migration. The low Pyro-P content in ice, often used in industrial catalysts or phosphate fertilisers, may be due to its structural properties, hindering its migration. Comparing the two lakes, Dai Lake exhibits a higher P content across all components than Wu Lake, which is consistent with the TP results (Dai Lake > Wu Lake). Moreover, Dai Lake shows multiple peaks for mono-P components, including inositol hexaphosphate, glucose-1-phosphate, and inositol cyclohexanol, as supported by previous studies [39,40,41].

From Figure 4, the concentration of each P component can be calculated based on the peak values of the ^31^P NMR spectra, using a 0.1 mg/L Ortho-P standard solution as a reference. Table 1 presents these results. Mono-P components were detected in the water samples but were absent in the ice samples, except for sample W4. In the Dai Lake water samples, the mono-P peaks reveal the presence of adenosine mono-P, with glucose-1-phosphate [42,43] being the predominant component, accounting for 78% of the total mono-P concentration. Other studies have shown that multiple chemical shifts in the mono-P peak may be related to the presence of various phosphate groups in phytic acid [44]. The concentration of Ortho-P in Dai Lake ice was significantly lower than in the water, whereas the Ortho-P concentration in Wu Lake ice was similar to that in the water. These suggest differences in P migration dynamics between lakes with different background values.

Among the mono-P components, adenosine monophosphate, a nucleotide found in RNA, indicates a link to biological metabolic activity [45]. Glucose-1-phosphate, a product of glucose phosphorylation, relates to the glucose content [40]. Phytic acid, derived mainly from crops such as corn and oats, suggests an influence from surrounding farmland drainage into Wu Lake [41]. Identifying these substances aids in understanding P sources and provides standard materials for future simulation experiments.

### 3.4. Simulation on the Change of P Concentration during the Ice–Water Phase Transition Process in Lakes

Four standard reference materials were identified based on the ^31^PNMR analysis results. Simulation experiments were conducted to study changes in P concentration during the ice–water phase transition in the lakes. The selected standard reference materials were a mixed phosphate solution, sodium Pyro-P, phytic acid solution, and triphenylmethyl phosphorus solution, representing Ortho-P, Pyro-P, and mono-P, respectively. The latter two substances were different components of mono-P. Triphenylmethyl P solution was added to macromolecular P substances to explore the changes in the concentration of P during the ice–water phase transition process.

**Figure 5:**
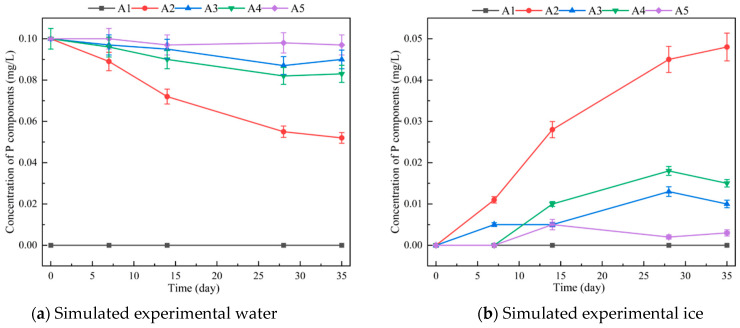
Dynamic changes of different phosphorus components with time in the ice–water simulated system (A1: blank sample; A2: Ortho-P standard solution; A3: sodium pyrophosphate; A4: inositol hexaphosphate; A5: triphenylmethyl phosphine).

The concentration-time curve of the simulation experiment is shown in Figure 5. During the freezing process of the lakes, phosphate is gradually repelled from the ice, whereas Pyro-P experiences greater repulsion due to its complex molecular structure, resulting in a lower overall concentration. Triphenylmethyl P was almost entirely excluded from the ice during the phase transition. This exclusion may be due to the large molecular structure of its benzene-ring-containing side group, causing strong crystal repulsion. These results indicate that during the ice–water phase transition, phosphate experiences the smallest repulsion, whereas mono-P and macromolecular P components are repelled to a greater extent.

**Figure 6:**
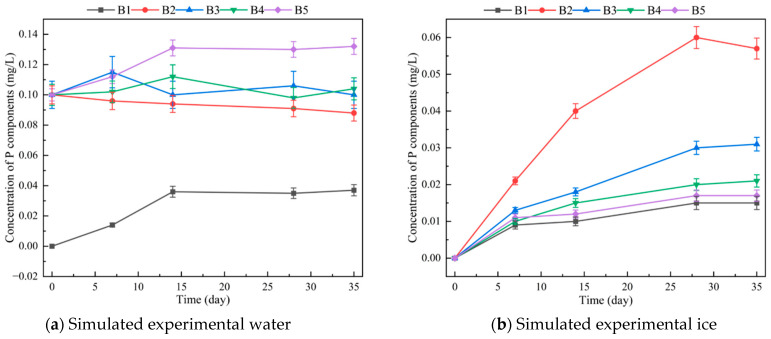
The dynamic changes of different phosphorus components with time in the ice–water–sediment simulated system (B1: blank sample; B2: Ortho-P standard solution; B3: sodium pyrophosphate; B4: inositol hexaphosphate; B5: triphenylmethyl phosphine).

To further simulate changes in P concentration during the freezing process, original lake sediment was added to the first simulation to explore the influence of sediment or microbial metabolism on P concentration changes during the phase transition. The TP concentration–time curve after adding sediment is shown in Figure 6. As shown for the blank group, P-containing substances released from the sediment gradually migrated into the water over time. Compared to the simulation experiment without sediment, the addition of sediment increased the P concentration between ice and water. Furthermore, the metabolic activity of microorganisms in the sediment may provide energy for the P migration process. Microorganisms in the sediment also influence migration between the ice and water interface by metabolising P-containing substances, leading to the decomposition of large molecules and reduced repulsion. However, the addition of sediment did not change the overall repulsion of large P molecules during the freezing process. This suggests that the main factor limiting P concentration during the ice–water phase transition is the molecular structure of P.

## 4. Discussion

### 4.1. Physical and Chemical Properties of Po in Environmental Media

The physicochemical properties of Po in ice-water sediments and other media differ significantly from those of other P components. Po is involved in photochemical reactions, where the steady-state concentration of hydroxyl radicals ·OH in sediment resuspension increases with the lake’s nutrient status. The higher the degree of eutrophication, the stronger the steady-state concentration of ·OH produced during sediment resuspension. Dissolved organic matter, nitrate, and Fe^3+^ are the main photosensitive substances that produce ·OH, releasing more dissolved phosphate and accelerating the eutrophication process [27]. Under alkaline conditions, Po undergoes photolysis and hydrolysis, releasing significant amounts of bioavailable P [3,29]. In addition, a significant correlation exists between Po components and humus-bound Po, indicating a strong interaction between Po and minerals. The rapid decomposition of DiP increases Po accumulation, and under the action of alkaline phosphatase, Po transforms into dissolved active P, especially in lakes with high algal biomass, thus accelerating metabolic processes [20]. Moreover, minerals can inhibit the adsorption of Po. Owing to the difficulty of enzymatic hydrolysis of phytate P, the interaction between Po and minerals in sediments is crucial for determining the biogeochemical cycle of Po in lakes. These nutrient states are closely linked to increases in organic pollution load and phytoplankton sedimentation [46]. Microorganisms also play a vital role in the chemical transformation of Po in lake environments. Their genetic diversity and ability to regulate gene expression enable them to adapt to harsh environments and participate in the P cycle under various conditions [16]. When microorganisms possess appropriate biochemical pathways and can cleave stable carbon–phosphorus bonds, Po can serve as an alternative source of Pi [3,47].

### 4.2. Qualitative Characterisation of the Chemical Composition of Po

The proportion of Po to TP in water and ice in the three lakes was higher than 50% (Figure 2a,b), especially in the winter, when the proportion of Po to TP was higher than that in the summer (Figure 2c), indicating that Po was the main component of TP. Via simulation experiments, Po components, such as inositol hexaphosphate and triphenylmethyl phosphine, migrate from water to ice (Figure 5 and Figure 6). Although not as fast and concentrated as Pi, such as Ortho-P, migrating from water to ice, Po components still migrate to ice during the freezing process. This migration and transformation process in the freezing and thawing cycle was an effective means for material circulation and energy conversion.

Recent advancements in Po research have led to the development of new technologies and methods for characterising its composition and structure [46]. One-dimensional liquid ^31^P NMR spectra are challenging to interpret due to overlapping signal peaks from similar phosphate groups. To address this, standard samples are added to correct the ^31^P NMR chemical shift of P (i.e., Ortho-P standard solution, sodium pyrophosphate, inositol hexaphosphate, and triphenylmethyl phosphine) (Figure 5 and Figure 6). Deconvolution analysis is then performed to identify and characterise up to 22 types of Mon-P components [3]. However, overlapping peaks remain a challenge, and spiking experiments alone cannot resolve all P components. The proportions of unknown Mon-P ranged from 9.7 to 24.2% [26].

To overcome these limitations, high-resolution two-dimensional hydrogen–phosphorus NMR spectroscopy (2D^1^H-^31^P NMR) has been used to identify and characterise Mon-P in soil samples. This method spreads NMR absorption signals across two separate frequency coordinates (H and P spectra), creating a two-dimensional NMR plane diagram of chemical shifts. This approach is more intuitive, clear, and reliable for the molecular structure characterisation of complex Mon-P compounds. Despite advancements, the detailed molecular structure characterisation of Po components needs improvement [46].

Incorporating 2D^1^H-^31^P NMR technology into Po studies in lake water environments, along with high-extraction, high-resolution, and non-destructive sample preparation techniques, can provide accurate and reliable qualitative and quantitative analysis results. This will ensure the characterisation and identification of Po composition across various water environments, laying the foundation for further research on the interfacial behaviour and migration processes of Po in multiple media.

Fourier transform ion cyclotron resonance mass spectrometry (FT-ICR MS) can also characterise substances at the molecular level [48]. FT-ICR MS is used for accurate mass determination, molecular formula calculations, and structural predictions of environmental samples, lake natural organic matter, proteins, and other biological macromolecules. It has broad applications in environmental sciences [49,50]. Applying high-resolution 2D^1^H- ^31^P NMR and FT-ICR-MS to study unknown chemical components in Po is crucial for understanding their biogeochemical cycling processes. This approach, combined with the characteristics of different media in lake water environments and optimised pre-treatment analysis methods and detection techniques, will enhance our understanding of Po’s role in these ecosystems.

### 4.3. Environmental Behaviour of Po Components Affected by Environmental Factors

The chemical properties and interfacial behaviour of sedimentary Po are influenced by environmental conditions such as land input and runoff characteristics. Analysis of the spatial distribution of Po in sediments using redundancy analysis and geochemical index methods over a 4000-year timescale revealed that precipitation and temperature, driven by latitudinal zonality, affect Po accumulation [51]. In situ experimental studies show significant seasonal variations in active phosphorus and Po in different lake regions, with aquatic plants acting as “pumps” in the P biogeochemical cycle. Human interventions, like harvesting aquatic plant debris, are crucial for controlling eutrophication in freshwater ecosystems [52].

The distribution of Po in sediment profiles also plays a key role in the P cycle [53]. Seasonal variations affect the migration and transformation of Po. For example, in the Grand River, during the winter and spring, Po continuously provides bioavailable P to lake water rather than Pi [54]. Additionally, endophytic detritus in lakes is a major contributor to Po pollution in sediments, with humus-bound HA-Po and humic-acid-bound Po being significant active Po forms in sediments [20]. The distributions of Po in suspended matter and cyanobacteria are similar to each other and differ from that in surface sediments. Combining solid-state ^31^P-NMR with liquid ^31^P-NMR revealed that several Po compounds associated with metals and other minerals could not be extracted from suspended matter and surface sediments. In Dian Lake, cyanobacteria are dominated by Po, poly-P, and Pyro-P, which can be released into lake water through chemical and biological degradation, exacerbating eutrophication [55].

Microorganisms play a pivotal role in the biogeochemical cycle of P in lake ecosystems. Both natural and human activities influence lake microorganisms, and the structure and diversity of microbial communities can reflect lake ecosystem functions and processes. High-throughput sequencing technology allows for a more accurate and comprehensive study of microbial community complexity and diversity, especially for microorganisms that are difficult to cultivate and have low abundance.

### 4.4. Biogeochemical Characteristics of Po at Multi-Media Interfaces

Currently, numerous reports address the interfacial effects and migration of P components, mainly focusing on the binding forms and interfacial migration activities of Pi [56]. However, few studies examine the interfacial migration behaviour of Po in lake ice, water, and sediment media. The interfacial behaviour of Po in lake water environments includes oxidation–reduction, adsorption–desorption, suspension–settlement, and degradation–complexation. These processes interact strongly with organic matter, minerals, heavy metals, and toxic particulates. Environmental conditions also significantly affect these interfacial behaviours. For example, a conceptual model of P behaviour shows that when salinity exceeds 6 ppt, significant desorption of P occurs, with Po desorption being greater than that of Pi. Phosphorus degradation remains the most important regeneration pathway for P in lakes [23,57].

Under high-salinity stress, HCl-P dominates in sediments, whereas under sediment dredging conditions, NaOH-P dominates. NaOH-P and HCl-P, mainly from human activities, dominate under different conditions. In situ organisms and human inputs are crucial sources of Po components, and sediment dredging enhances the release of Po into the overlying water [58]. Additionally, extracellular enzymes accelerate the release of P components during dredging, leading to anaerobic conditions in lake water [59].

In summary, the molecular structure and migration behaviour of Po components in different media in cold and arid lake regions remains unclear. More analysis is needed on the interfacial behaviours during ice-covered periods, in water, and in sediments. The contribution of endogenous P to eutrophic lakes and its impact on water blooms are not yet fully understood, significantly limiting the study of the biogeochemical cycle of Po in lake water environments. Future research should focus on analysing interfacial behaviours during ice-covered periods, in water, and in sediments to better understand these processes and their environmental impact.

## 5. Conclusions

The study found that the P content in the ice of the three lakes during winter was lower than in the water, with Po accounting for 70% of the TP in both ice and water. Among the three lakes, Dai Lake had the highest P content in winter, while Wu Lake had the lowest TP content. In Wu Lake, the TP content in the summer was approximately five times higher than in the winter, indicating a high potential for eutrophication despite the lower winter P content due to the high proportion of bioavailable P components.

Other nutrient parameters, such as TOC and TN, were also analysed to assess the degree of eutrophication in the lakes. It was observed that Dai Lake’s eutrophication in the winter is mainly controlled by P sources. Influxes of P during the spring can lead to algal blooms. Conversely, Wu Lake is influenced by nitrogen and P sources during the winter, suggesting a significant potential for eutrophication. As ice melts in the spring, the influx of exogenous nitrogen and P can lead to algal blooms and other eutrophication phenomena.

The results from ^31^P-NMR indicated that the P components in the water were primarily Ortho-P and Mon-P, accounting for more than 90% of the TP. The content of Mon-P and Pyro-P in ice was relatively low, indicating that during the freezing process, the intermolecular repulsion caused by the phase change increased. This strong repulsion against large molecules such as Mon-P results in their low content in ice.

The simulation experiment results confirmed that the formation of ice crystals during the phase transition process exerts a repulsive effect on P components. Larger molecular structures of P components experience greater repulsive forces during the phase transition, resulting in a significantly higher distribution of P components in water than in ice.

The findings highlight the dynamic nature of nutrient distribution between the ice and water phases and its implications for eutrophication. The higher concentration of bioavailable phosphorus in water during the winter and its potential increase in the summer suggests a need for careful monitoring and management to prevent algal blooms and maintain water quality. The study also underscores the importance of considering seasonal variations in nutrient management strategies.

Future research on P in cold, arid lakes will identify sources and monitor migration, focusing on seasonal impacts. The limitations of this study include sampling difficulties and model accuracy, necessitating long-term data accumulation. The mechanisms show cross-climatic commonalities, emphasising the value of research across lakes. The methods also apply to rivers and estuaries, offering new insights into P cycles and eutrophication control. This research is crucial for combating eutrophication and protecting lake ecosystems and has broad application potential.

## Figures and Tables

**Table 1:** Concentrations of P components by ^31^P NMR in the ice and water of the lakes.

Samples	Ortho-P (mg/L)	Mon-P (mg/L)		Pyro-P (mg/L)
		Adenosine Monophosphate	Glucose-1-Phosphate	
DH2 water	0.034	0.002	0.011	0.005
DH3 water	0.021	0.002	0.003	0.003
W4 water	0.013	——	0.002	0.003
W6 water	0.010	——	0.003	0.002
DH2 ice	0.007	——	0.003	0.001
DH3 ice	0.005	——	——	——
W4 ice	0.017	——	0.013	0.001
W6 ice	0.010	——	——	——

## Data Availability

The datasets used or analysed during the current study are available from the corresponding author upon reasonable request.

## Supplementary Materials

The following supporting information can be downloaded at: https://www.mdpi.com/article/10.3390/toxics12070523/s1, Table S1: Atmospheric and water temperatures of three lakes; Table S2: Extraction efficiency of phosphorus; Table S3: TOC, TN, and TP and their ratios from lakes.
