# Supplementary material for: Forms and Migration Mechanisms of Phosphorus in the Ice, Water, and Sediments of Cold and Arid Lakes
Source: Toxics. 2024 Jul 20;12(7):523. doi: 10.3390/toxics12070523 (PMC11280900; doi:10.3390/toxics12070523)
Supplement: Supplementary file 1 [file toxics-12-00523-s001.zip › toxics-3096443-supplementary.pdf]

Support information

Table S1. Atmospheric and water temperatures of three lakes.

Sampling time	Lakes	Atmospheric temperature (℃)	Water temperature(℃)
January 12, 2022	Dai Lake	-8 ~ -19	-0.13 ~ 0.55
	Wu Lake	-3 ~ -16	-0.84 ~ -1.03
	Hu Lake	-21 ~ -32	-1.05 ~ -1.86
August 3, 2022	Dai Lake	32 ~ 19	13.3 ~ 19.6
	Wu Lake	33 ~ 21	14.5 ~ 20.2
	Hu Lake	31 ~ 17	12.8 ~ 18.4
Data sources	Dai Lake	https://www.tianqi24.com/wulanchabu/history.html	Measured data
	Wu Lake	https://www.tianqi24.com/bayanzhuoer/history202208.html	Measured data
	Hu Lake	https://www.tianqi24.com/hulunbeier/history.html	Measured data

Table S2. Extraction efficiency of phosphorus

Type	Samples	Concentration of P before extraction (mg/L)	Concentration of P after extraction (mg/L)	Extraction efficiency (%)
Water	W4	0.041	0.024	58.54
Water	W6	0.048	0.027	56.25
Water	DH2	0.074	0.052	70.27
Water	DH3	0.065	0.039	60.00
Ice	W4-ice	0.028	0.015	53.57
Ice	W6-ice	0.029	0.017	58.62
Ice	DH2-ice	0.062	0.028	45.16
Ice	DH3-ice	0.057	0.031	54.39

Table S3. TOC, TN, and TP and their ratios from lakes

Samples	Type	TOC (mg/L)	TN (mg/L)	TP (mg/L)	TOC/TP	TN/TP
W1	Water	10.16	5.58	0.090	112.89	62.00
	Ice	4.58	1.55	0.035	130.86	44.29
W2	Water	14.03	0.78	0.059	237.80	13.23
	Ice	0.941	0.55	0.036	26.14	15.28
W3	Water	8.36	0.62	0.053	157.74	11.70
	Ice	1.35	0.39	0.037	36.49	10.54
W4	Water	7.42	0.44	0.062	119.68	7.10
	Ice	2.15	0.16	0.044	48.86	3.64
W5	Water	2.32	0.25	0.050	46.40	5.00
	Ice	1.26	0.10	0.043	29.30	2.33
W6	Water	15.36	0.75	0.063	243.81	11.90
	Ice	1.64	0.42	0.027	60.74	15.56
W7	Water	7.29	0.16	0.076	95.92	2.11
	Ice	2.69	0.09	0.028	96.07	3.21
DH1	Water	74.51	10.28	0.126	591.35	81.59
	Ice	18.57	4.28	0.033	562.73	129.70
DH2	Water	81.37	11.35	0.158	515.00	71.84
	Ice	23.94	2.56	0.072	332.50	35.56
DH3	Water	60.42	9.83	0.133	454.29	73.91
	Ice	27.84	1.35	0.077	361.56	17.53
DH4	Water	60.30	10.25	0.140	430.71	73.21
	Ice	24.70	5.56	0.025	988.00	222.40
DH5	Water	74.47	15.25	0.149	499.80	102.35
	Ice	21.43	3.32	0.023	931.74	144.35
DH6	Water	55.91	14.31	0.126	443.73	113.57
	Ice	27.46	4.42	0.024	1144.17	184.17
DH7	Water	74.53	13.37	0.134	556.19	99.78
	Ice	18.23	6.65	0.074	246.35	89.86
DH8	Water	53.96	13.42	0.130	415.08	103.23
	Ice	25.53	7.50	0.081	315.19	92.59
DH9	Water	34.77	10.35	0.147	236.53	70.41
	Ice	11.90	4.25	0.090	132.22	47.22
